# An evaluation of intervention appropriateness from the perspective of parents: A peer‐mediated, play‐based intervention for children with ADHD

**DOI:** 10.1111/1440-1630.12981

**Published:** 2024-07-20

**Authors:** Sarah Wilkes‐Gillan, Lauren Parsons, Dave Parsons, Natasha Mahoney, Nicola Hancock, Reinie Cordier, Michelle Lincoln, Yu‐Wei (Ryan) Chen, Anita Bundy

**Affiliations:** ^1^ School of Health Sciences, Faculty of Medicine and Health The University of Sydney Sydney New South Wales Australia; ^2^ Curtin School of Allied Health, Faculty of Health Sciences Curtin University Perth Western Australia Australia; ^3^ Department of Social Work, Education and Community Wellbeing, Faculty of Health and Life Sciences University of North Umbria Newcastle upon Tyne UK; ^4^ Faculty of Health University of Canberra Canberra Australian Capital Territory Australia; ^5^ Department of Occupational Therapy, College of Health and Human Sciences Colorado State University Fort Collins Colorado USA

**Keywords:** ADHD, friendships, intervention, parents, play, social skills

## Abstract

**Introduction:**

A peer‐mediated, play‐based intervention has been developed to address social participation challenges experienced by children with ADHD. To facilitate implementation into clinical practice, interventions should be evaluated for appropriateness to the end‐user, as well as effectiveness. Previous research demonstrated the approach is effective for improving children's social play skills. This study aimed to evaluate the appropriateness of the intervention for children with ADHD and their families.

**Methods:**

Parents of children with ADHD who participated in the play‐based intervention were interviewed 1 month after completion. Parents were asked about their perspective of parent and children's experiences of the intervention, the perceived benefits for children and parents, the logistics of participating, and recommended adaptations to the intervention. Interviews were analysed thematically, and themes were mapped to the elements of the adopted definition of appropriateness to understand whether parents supported the appropriateness of the intervention for their families.

**Consumer and Community Involvement:**

Consumers were not directly involved in the decisions made about this study.

**Findings:**

One core theme, ‘collaborative efforts’, emerged from the data. Major themes comprising the core theme were ‘on the same page’, ‘therapeutic relationship’, and ‘getting the job done’. Three sub‐themes of ‘engagement and motivation’, ‘the effort was worth it for the reward’, and ‘Rome wasn't built in a day’ were nested within the major themes.

**Conclusion:**

Parents largely supported the appropriateness of the intervention, indicating it addressed an important goal for their child, participation was a positive experience, and they perceived the intervention to be beneficial. Future adaptions of the intervention are needed to increase its ecological validity and to generalise the strategies to other social environments and playmates, such as peers at school.

**PLAIN LANGUAGE SUMMARY:**

This study looked at an intervention that uses play with peers to help children with ADHD develop their play skills. The researchers wanted to know if parents thought the intervention was a good fit for their families and if it helped their children. Parents gave an interview a month after the intervention ended. They were asked about their thoughts on the intervention, how it helped their children and themselves, how easy it was to take part, and what changes could be made to make the intervention better. After analysing parents' answers, the researchers found parents mostly agreed that the intervention was a good fit. They said it helped their children to play with their peers, and they had a good time doing it. Parents thought the intervention was helpful, they liked that it was a joint effort between them and the occupational therapist, and they felt it was worth the effort. However, they also suggested some changes. They wanted the intervention to fit into other real‐life social situations, such as school, so their children could use the skills they learned in other places. Overall, parents thought the intervention was helpful and suitable for their children and themselves, but some changes might make it more helpful.

**Key Points for Occupational Therapy**

- Parents play a key role in supporting their child's peer interactions and friendship development.
- The play‐based intervention was a positive experience, beneficial and relevant/important from parents' perspectives.
- Ecological validity of the intervention would be enhanced through delivery in the child's real‐life settings such a school.

## 1 INTRODUCTION

Children with attention deficit and hyperactivity disorder (ADHD) commonly experience difficulties with inattention, impulsivity, and hyperactivity (American Psychiatric Association, 2013; Fabiano et al., 2021). Due to these challenges, they often develop poorer peer relationships than their neurotypical peers (Ros & Graziano, 2018). Poor peer relationships in childhood have been associated with significant downstream effects in adult life, such as psychiatric disorders, lower incomes, and a high chance of anti‐social behaviours (Bishop et al., 2019; Hechtman et al., 2016; Pelham et al., 2020), necessitating the development of interventions that can enhance peer relationships.

Many studies have evaluated the effectiveness of psychosocial interventions for supporting the play, peer involvement, and social functioning of children with ADHD. Systematic reviews report mixed findings for effectiveness among these interventions (e.g., Cordier et al., 2018; Cornell et al., 2018; Morris et al., 2021), with some interventions leading to greater intervention effects than others. Among these studies is a body of evidence dedicated to developing and evaluating a play‐based, peer‐mediated intervention targeting the social play skills of children with ADHD. First, a model was developed detailing interactions between the traits of ADHD and factors that promote play, with a set of principles to underpin a play‐based intervention (Cordier et al., 2009). Next, therapist‐delivered and parent‐delivered iterations of the intervention were piloted in small, single group studies, and evaluated for feasibility, preliminary effectiveness, and appropriateness (Wilkes‐Gillan et al., 2014; Wilkes‐Gillan, Bundy, Cordier, & Lincoln, 2016; Wilkes‐Gillan, Bundy, Cordier, Lincoln, et al., 2016; Wilkes‐Gillan et al., 2011). Finally, a randomised controlled trial (RCT) evaluated the effectiveness of the intervention; finding improvements in the social play skills of children with ADHD and their typically developing peers upon completion of the intervention (Wilkes‐Gillan, Bundy, Cordier, Lincoln, et al., 2016; Wilkes‐Gillan et al., 2022). Despite the existence of effective psychosocial interventions for children with ADHD, such as this, the translation of psychosocial interventions from research settings to practice settings seems to be lacking.

The successful implementation of, and engagement by families with, psychosocial interventions for children with ADHD is poorly understood (DuPaul et al., 2020; Malti et al., 2016). For example, one study in the United States reported that only 31% of families of children with ADHD received psychosocial interventions, while 91% received psychotropic medication (Danielson et al., 2018). Furthermore, if psychosocial interventions are accessed by families, interruptions and abandonment rates with psychosocial interventions are often high (Baweja et al., 2021). Systemic barriers (e.g., availability of services and financial) may lead to the discontinuation of psychosocial interventions by families, but parent attitudes, perceptions, and preferences are critical factors that can facilitate intervention engagement (Baweja et al., 2021; DuPaul et al., 2020). Adopting a systematic approach, that involves end‐users, to the design and evaluation of interventions has utility in addressing implementation challenges.

To ensure interventions are able to provide solutions to real‐world issues, in addition to effectiveness, they need to be implementable, appropriate to the end‐users, feasible, scalable, and transferable across differing contexts (Skivington et al., 2021). The United Kingdom Medical Research Council (UKMRC) devised a framework, providing a stepwise, phased methodology for designing and evaluating complex interventions (Skivington et al., 2021). The framework comprises four stages that can be used iteratively or sequentially: (1) development or identification, (2) feasibility, (3) evaluation, and (4) implementation. Critical to intervention evaluation, the framework acknowledges that most complex interventions are evaluated from an efficacy or effectiveness perspective, and utilising a wide range of research perspectives is critical to evaluate interventions beyond efficacy or effectiveness (Lyon & Koerner, 2016; Skivington et al., 2021). Given the apparent challenges in the implementation of psychosocial interventions for children with ADHD, understanding the end‐user experience may provide useful insights to bridge the gap between evaluation and implementation.

One way to investigate the end‐user experience within the context of intervention development is to evaluate the appropriateness of an intervention. Evans (2003) defines appropriateness as the impact of the intervention from the perspective of the end‐user. The appropriateness of an intervention is concerned with the experience of the end‐user, issues important to the end‐user, and whether the end‐user views the outcomes as beneficial (Evans, 2003). Moreover, interventions should be appropriate for the end‐user to improve the implementation of the intervention into clinical practice. Failure to consider the end‐user by intervention designers could result in undesirable consequences such as incorrect implementation, delays in adoption, or rejection of the intervention entirely (DuPaul et al., 2020; Vivanti et al., 2018). In the context of the aforementioned play‐based, peer‐mediated intervention, parents can be considered a vital end‐user given their role in engaging with the therapy providers, organising logistics related to the intervention (i.e., transportation, coordinating peers), and facilitating parent home‐based tasks. Moreover, parents have a key role in supporting their child's peer interactions and friendship development and, therefore, are integral in the support of their children's play (Waldman‐Levi & Bundy, 2016). As such, obtaining parent's perspectives regarding the appropriateness of the intervention is essential.

This study aimed to evaluate the appropriateness of the play‐based, peer‐mediated intervention for children with ADHD. The RCT by Wilkes‐Gillan, Bundy, Cordier, Lincoln, et al. (2016) provided evidence of the effectiveness of the intervention, and the next step in the development of this intervention approach is implementation into occupational therapy practice. A framework previously employed to understand appropriateness in intervention trials with similar populations was adopted for this study (Allan et al., 2018; D. Parsons et al., 2019; Wilkes‐Gillan et al., 2015). The framework comprises of five domains: (1) the participant perceives the intervention addresses a relevant or important issue, (2) the participant has a positive experience, (3) the intervention has beneficial outcomes, (4) the invention's components are viable in the participants everyday context (ecologically valid), and (5) the strategies developed through the intervention are continued once the intervention is ceased. As parents are important stakeholders in their children's social participation and end‐users of this intervention, their perspectives on the intervention's appropriateness are therefore critical to its evaluation. Findings from this study are anticipated to provide insights into the design of ongoing improvements to the intervention and inform strategies to further facilitate its implementation.

## 2 METHODS

This qualitative study took a phenomenological approach. Reflexive thematic analysis was used to analyse semi‐structured interview responses from parents of children with ADHD who participated in the RCT of the play‐based intervention (Braun & Clarke, 2006, 2019). Prior to conducting the study, ethical approval was obtained from The University of Sydney's Human Research Ethics Committee (approval 2013/109).

### 2.1 Author positionality

This study's research team comprised individuals with multidisciplinary professional backgrounds, including occupational therapy, psychology, and speech pathology. The authors have a commitment to improving the social participation of children with ADHD and poses collective diversity of experiences as both clinicians and researchers in their respective fields. In implementing this study, the research team recognised the importance of reflexivity in acknowledging how individual identities and experiences may have influenced interactions with research participants and the interpretation of data.

### 2.2 Participants

Purposive sampling was used to invite parents of children with ADHD who participated in the play‐based intervention RCT to participate in this study. Parents were eligible to participate if they had attended the intervention sessions with their child and were available for an interview 1 month following the completion of the intervention. All parents involved in the RCT were eligible, and therefore, all parents were invited to participate in an interview by the first author at the 1 month follow‐up data collection point. Those who agreed to participate provided written consent. The intervention was delivered across 2013 and 2014, with follow‐up interviews competed as of October 2014.

### 2.3 Intervention procedures

The intervention delivered in the RCT involved children with ADHD and a typically developing peer attending six clinic‐based play sessions with an occupational therapist across 8 weeks and home‐based tasks facilitated by parents. Clinic‐based sessions involved self‐modelling of play skills through video feedback and feedforward and therapist and peer modelling through child‐led free play. Parents of children with ADHD attended the sessions to observe the video feedback and therapist modelling, discuss their child's play skills with the therapist, and develop strategies to implement at home. Parents completed manualised tasks at home with their child each week. The chapters of the parent manual focussed on social skills or contexts children may find challenging (e.g., dealing with competition and reading social cues). Chapters were prescribed to parents based on therapist observations of play in clinic play sessions and baseline play skills. The home tasks involved parents reading a manual chapter about a particular play skill or challenge and then viewing a prerecorded video with their child and facilitating a discussion about the play and strategies they observed through the characters in the video. Parents also arranged a playdate for the dyad in the weeks between clinic sessions so that children had opportunities to apply the strategies and skills modelled in the clinic sessions and discussed with parents during home‐based tasks.

### 2.4 Interview schedule

A semi‐structured interview schedule was developed to explore the appropriateness of the intervention. The schedule contained open‐ and close‐ended questions, guiding the interviewer to explore the five components of appropriateness adopted for the study. Questions related to participation in the intervention, focussing on why families initially came to participate in the intervention, parent and child experiences of the intervention at the clinic and at home, derived benefits for children and parents, the logistics of participating, and recommendations for changes or adaptations to the intervention. The interviewer also asked parents to rate their experience and perceived benefits on a 10‐point scale (e.g., 10 = maximum enjoyment and benefits) as a means of summarising parents' overall responses and to prompt further questions if ratings were incongruent with the narrative for any given area.

### 2.5 Interview procedures

A researcher not involved in the delivery of the intervention conducted the interviews with each parent via telephone. Interviews lasted 40–60 minutes and were recorded using a digital audio recorder. Following the interviews, all audio files were transcribed verbatim.

### 2.6 Data analysis

Interview transcripts were then analysed thematically using the constant comparative method by Strauss and Corbin (2004) and guided by Braun and Clarke's (2006, 2019) reflexive thematic analysis. Transcripts were initially coded line by line using open codes, comparing and categorising data. Axial codes were then created to link together open codes that related via context, topic, or action. Finally, selective coding was used to create sub‐themes and overarching themes that best described phenomena found in the data (Strauss & Corbin, 2004). The final set of themes were then mapped against the adopted appropriateness framework to understand the relationship between the themes as either supportive or unsupportive evidence for the appropriateness of the intervention. A theme was mapped to a domain within the framework if its content was aligned with the definition of the domain. To ensure trustworthiness of the findings, two authors independently coded the same three transcripts and met to discuss agreement of the coding, review themes, and reach consensus. Once agreement was reached, one author coded the remaining transcripts, meeting regularly with two authors to ensure trustworthiness, consistency, and limit bias. Mapping of the themes to the appropriateness framework was completed by one author and cross checked with two co‐authors for agreement. Discrepancies in agreement were discussed until a consensus was reached.

## 3 FINDINGS

### 3.1 Participants

All 25 parents of children with ADHD who participated in the intervention completed an interview. Demographic information for participants can be found in Table 1.

**TABLE 1:** Participant demographics.

Parents^a^	
Mean age (SD)	41.6 (6.50)
Born in Australia	18 of 25
Education: Degree or diploma	90%
Occupation: Requires tertiary qualifications	58%

Children with ADHD	
Mean age (SD)	8.3 (1.6)
Male	25 of 29
Born in Australia	26 of 29

^a^ Number of parents is not equal to number of children with ADHD, as some parents enrolled more than one child in the study.

### 3.2 Interview themes

**FIGURE 1:**
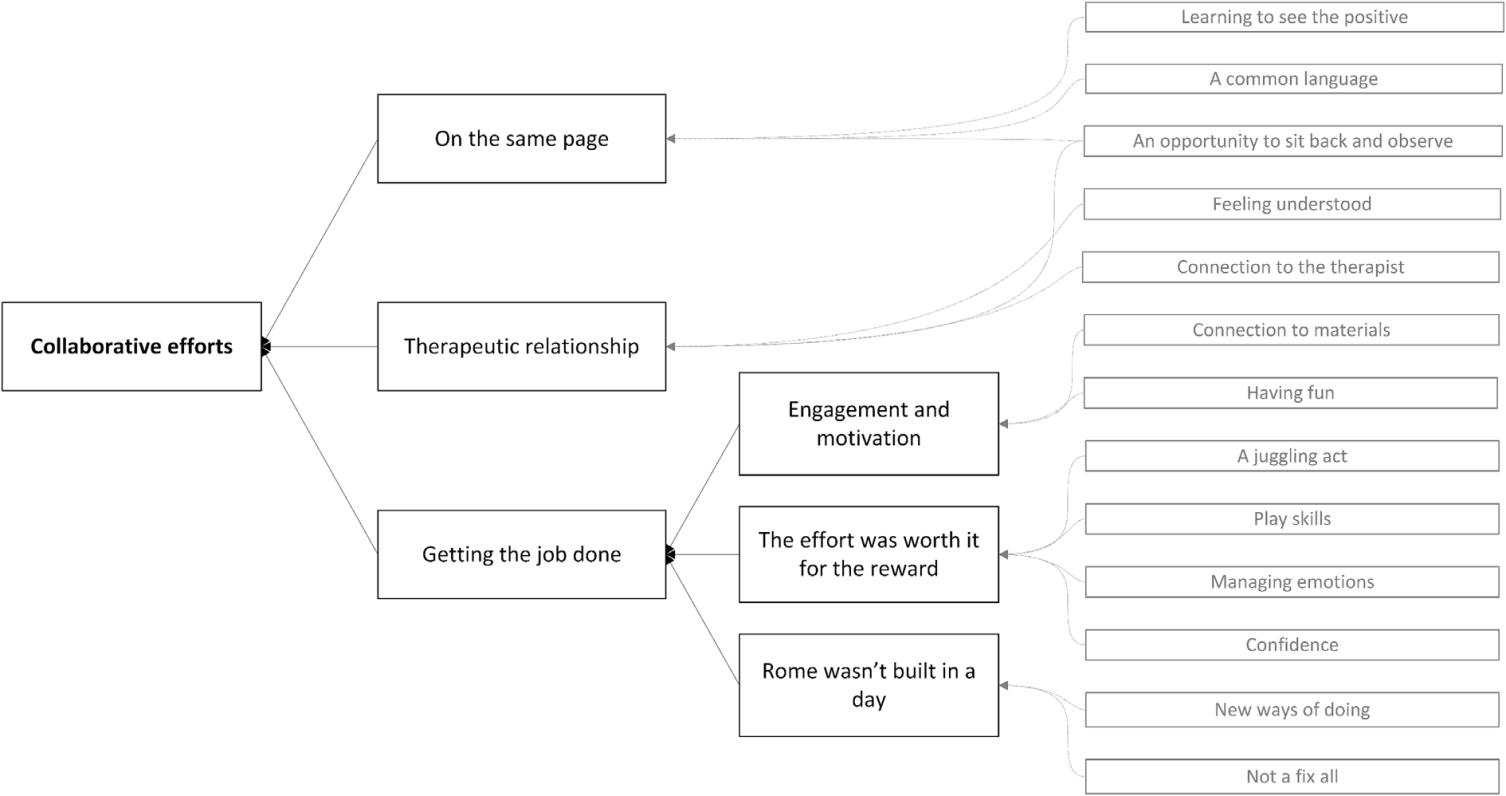
Development of final framework.

Open coding of the 25 transcripts resulted in 231 initial codes. These codes were grouped together to develop an initial set of 13 themes. These initial themes were reviewed and collapsed into a theme framework consisting of a single core theme, three major themes, and three sub‐themes. Figure 1 demonstrates how the initial themes were collapsed into the final theme framework described below.

#### 3.2.1 Core theme: Collaborative efforts

Overall, parents expressed the intervention was beneficial to their children, participation was enjoyable, and made possible through the collaboration of the therapist, parents, and children. Everyone played a unique role in implementing the intervention, but those roles required dedication and a deliberate effort from all individuals involved to achieve the goal of the intervention for the children. Through observation, coaching, and collaboration with the therapist, parents became more expert observers of their child's play, and parents gained a clarity about how their child engaged in positive playful experiences with peers. The shared experience of participation between parent and child gave children and parents a common understanding of play and the strategies children can use to engage in positive social play with their playmate and peers away from the clinic.

##### Major theme: On the same page

Parents developed a new understanding of their child through observation of play sessions, describing a ‘reframing’ of their thinking. This change in perspective was attributed to the opportunity to sit back and observe their child play in the intervention sessions, combined with coaching and discussion with the therapist. Learning about their child's needs and behaviour through observation meant that parents could use this new awareness to support play time at home.

Many parents also noted the intervention gave children and parents a common language to talk productively about social play. This common language allowed parents to communicate with their child and give cues in terms that they both understood:

And the fact that there's been cues in this sort of manner have allowed both of us to have a common language to describe something both inside a structured situation and outside the structured situation. And so immediately, as soon as I use one of those cues he knows the world of information that I'm talking about.

The common language also allowed parents to reinforce intervention strategies discretely and without embarrassing or singling out their child in a play situation. Parents described being able to communicate more calmly with their child, because the common language meant they were able to be more intentional with their communication.

##### Major theme: Therapeutic relationship

Parents described the involvement of the therapist as critical to their own and their children's learning and enjoyment of the intervention. The empathy displayed by the therapist was appreciated by parents who described often feeling judged by other adults. Several parents expressed feeling the therapists deeply understood and cared about their child's needs, and interacted with their child in a non‐judgemental way, which contrasted with previous experiences with other professionals:

Well I was really happy with it and like when [lead researcher] first came out to the meeting I first went to, I think she said how much she loved kids with ADHD, she just had us all won over right then and there. Cos we just feel like you know? Teachers just find them all too hard, nobody really likes or understands them and she just melted all of our hearts I think when she just said that, it's just like ‘finally somebody that likes our kids’.

Mirroring their own experiences, parents described their children enjoying that the therapist ‘got on their level’ to play. For some children this was particularly novel, as it was rare for adults to play with them during the later stages of middle childhood. This connection with the therapist made the experience enjoyable and allowed children to feel understood. Parents also appreciated the opportunity to learn from someone with expertise, finding value in the evidence‐based nature of the intervention. Parents also reflected on learning by direct observation of the therapist implementing strategies within the playroom. While the therapist played a strong role in the learnings of both parents and children, some parents also suggested more therapist support was needed to generalise and apply the newly learnt strategies to range of social scenarios to optimise the intervention for families.

##### Major theme: Getting the job done

Parents spoke about the challenges their family overcame so their child could participate in the intervention and the factors that contributed to their engagement in the intervention process. On the whole, parents described the intervention as an enjoyable and beneficial experience, but participation took planning and resources to see the process through. The challenges described by families who committed to participating in the intervention were paired with a parallel sentiment that the challenges were not insurmountable and ‘worth it’ for the benefits their child received. Many of the intervention techniques were motivating to the children, which reduced the burden of participation, as parents could see their child was enjoying themselves. Parents were however cognisant that ongoing use of the strategies would be needed for development to continue and for strategies to be applied to different contexts.

*Sub‐theme: Engagement and motivation*. The video‐modelling videos were a source of anticipation and excitement for children at the start of each session, as they enjoyed seeing themselves on the screen and identifying instances of ‘red’ and ‘green’ play in real time. Seeing themselves on video allowed children to connect directly with the social challenges addressed through the intervention and made the concepts they were learning about more concrete. Almost all parents described their child enjoying the play sessions. The child‐led, free, and unstructured nature of the play with a peer was a particular source of fun, and having fun became the motivation for children to engage in and learn from participating in the intervention. Some children were initially reluctant to participate, as they were unsure of was involved, or were generally withdrawing from participating in most things. However, interactions with the therapist and the video feedback helped in overcoming this initial reluctance:

I think he started to get on board once he got to know, getting to know the people who were doing the intervention was really key. But important that he started to develop rapport with [therapist]. He seemed interested and engaged with the resources and I think particularly looking at his own play seems to be really important, seems to be a big factor.

*Sub‐theme: The effort was worth it for the reward*. Finding a playmate before starting the intervention was difficult for some families. Experiences of stigma from parents with typically developing children reduced the likelihood of discussing the intervention with other parents. In addition, some children did not have many friends to choose from, and the duration of the intervention period was long, relative to how long their friendships tended to last. Families faced with these challenges opted for a sibling or other family member to attend the intervention as a playmate. Getting to the clinic sessions, dealing with traffic, and fitting weekly clinic sessions into already busy schedules were common challenges. Despite the challenges of participating, parents overwhelmingly felt that participating in the intervention was worth the time, effort, and accommodations made to be there because of the tangible, observable benefits to their child.

The benefits to children noted by parents included changes in children's turn taking and sharing while playing with a peer were described by parents; a change attributed to their child's internalisation and subsequent application of the play concepts and strategies introduced through the intervention. Parents also described their children applying specific strategies that increased cooperation, and improvements in their child's empathy both during play with their peer, and in other settings such as school. Others described changes in their child's ability to regulate their own emotions, which some attributed to an increased ability to verbalise their frustrations, and a sense of empowerment when communicating their emotions to their peer.

A majority of parents noted that their child had become more confident, particularly in regard to approaching their peers. Parents attributed this increased confidence to their child's learnings about themselves during the intervention period. The intervention allowed children to feel ‘not so strange’ and showed them that they can participate in positive play interactions with peers.

I think become more confident in himself and, 'cause really, he was interested in making friends. That's what I've noticed now, he's approaching people … It's gone from being completely friendless walking around the playground with his hands in his pockets to having a really good best friend who he understands.

*Sub‐theme: Rome wasn't built in a day*. Parents recognised a need for continued use of intervention strategies after sessions with the therapist were complete to maintain and reinforce learning. Parents described themselves as being armed with new skills and commonly implemented strategies such as creating a clear play space at home, planning out playtime on whiteboards, planning more playdates, and re‐watching the program materials after the intervention period had ended. Most parents were able to use the intervention strategies with their child at home with relative ease, especially when playing involved the playmate who attended the intervention sessions. Strategies were more challenging to generalise to the school setting and play with other playmates. Some children were resistant to hearing about the intervention concepts in new settings, while others described that they did not always remember to implement the strategies in the moment with other playmates. Despite these challenges, parents reflected that the intervention gave them a frame of reference to return to beyond the cessation of clinic‐based sessions. Coupled with the notion that they will need to keep up with reinforcing the skills, almost all parents indicated they would also like a follow‐up session with the therapist to ensure the benefits are maintained and development continues:

A follow up, um three months later or four months later just to, and if possible bring back the friend and have another play and just see if they're still on track with the, their play if it's still, well green play than red play.

### 3.3 Mapping of themes to the appropriateness framework

**FIGURE 2:**
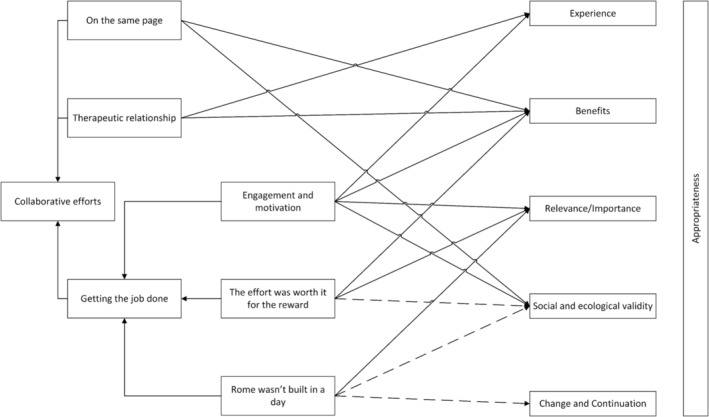
Connections between interview themes and components of intervention appropriateness.

The mapping of themes to the domains of appropriate identified that all domains of were associated with at least one theme. Figure 2 outlines the connection between themes and the adopted appropriateness framework, which is described forthwith.

The domain of appropriateness ‘Experience’ relates to end‐users having a positive experience while participating in an intervention. The themes ‘therapeutic relationship’ and ‘engagement and motivation’ were mapped to the Experience domain and were considered to be evidence supporting this element of appropriateness. The content of these two themes pointed to participation in the intervention being enjoyable and exciting for both parents and children due to the nature of the therapist involved and the playful components of the intervention.

‘Benefits’ as a domain of appropriateness is concerned with whether the intervention has beneficial outcomes for participants. Four themes were mapped to Benefits (‘therapeutic relationship’, ‘engagement and motivation’, ‘on the same page’ and ‘the effort was worth it for the reward’). These themes were evidence of positive support for appropriateness, as these four themes pointed to observable positive changes for families that parents associated with the experience of the intervention and its constituent components.

As a domain of appropriateness ‘Relevance/Importance’ relates to an intervention addressing a relevant or important issue for its end‐user. Three themes were mapped against ‘Relevance/Importance’: ‘engagement and motivation’, ‘the effort was worth it for the reward’ and ‘Rome wasn't built in a day’. These three themes were considered positive evidence for appropriateness as they related to families' commitment to the intervention, which occurred because parents and children enjoyed observing and participating in positive peer–peer play. Parents also recognised the need to continue to implement the intervention strategies in the long term and were doing so in recognition of the importance of promoting their children's play skills.

‘Social and ecological validity’ is concerned with the viability of an intervention's components within participants' everyday contexts. Partial support for Social and ecological validity was found within the themes, with themes were mapped to the domain: two (‘on the same page’ and ‘engagement and motivation’) acting as positive support for the domain and two (‘the effort was worth it for the reward’ and ‘Rome wasn't built in a day’) as unsupportive. Elements of the intervention, such as play and the language used, were easily integrated into families' lives, but the logistics of attending the clinic, findings a suitable playmate, and the limited social contexts (e.g., a single playmate, no group play, clinic environment, and home environment only) limited the ecological validity of the intervention and its outcomes for some families.

The domain of ‘Change and continuation’ is concerned with the continued use of strategies developed through an intervention once the intervention has ceased. ‘Rome wasn't built in a day’ was the only theme mapped to Change and continuation. The theme was considered to be partially supportive of this element of appropriateness, as some families continued to apply the strategies and skills developed through the intervention period but expressed a need for further support in the long term as their child continued to develop.

## 4 DISCUSSION

This study sought to evaluate the appropriateness of a novel play‐based peer‐mediated intervention targeting playfulness and pragmatic language for children with ADHD. A key aspect of the intervention was using children's primary occupation of play to provide an intrinsically motivating context to support skill development (Wilkes et al. 2011). A five‐component framework of appropriateness was adopted for the study, and appropriateness was evaluated from the perspective of parents of children with ADHD who participated in the 8‐week intervention (Allan et al., 2018; D. Parsons et al., 2019; Wilkes‐Gillan et al., 2015). Themes that emerged from interviews with parents supported three of the five aspects of appropriateness and partially supported the remaining components.

Beneficial outcomes were the component of appropriateness most strongly supported by parents. Parents described observable changes in their child's social play skills, the use of those skills by their child to start new friendships, and their own development of new understandings about their child and strategies learned through direct observation. The relationship developed between the therapist and the families appeared to be a prerequisite to the benefits. The benefits described by parents within this study mirror those described in pilot studies of the same intervention techniques for children with ADHD and autistic children. Parents of children with ADHD who participated in piloting of the intervention spoke of similar benefits for their child's play experiences and parents changing their perspectives of their child's play (Wilkes‐Gillan et al., 2015). Similarly, parents of autistic children described their children communicating more effectively to initiate and maintain play with peers, and the common language developed through the intervention resulted in a role shift for parents, from ‘umpire’ to ‘facilitator’ (L. Parsons et al., 2019). Parents' perceptions of the benefits after participating in this RCT also corroborate findings for the intervention's effectiveness where measurable changes in observable behaviours of playfulness were found for children with ADHD and their typically developing peers, particularly in aspects of playfulness related to social play (Wilkes‐Gillan et al., 2022).

The combination of intervention techniques and play‐based nature of the intervention contributed to the intervention as a positive experience for participants. Children were likely motivated by the play‐based approach to the intervention. Play is the primary occupation of children, and enjoyable, but importantly for the intervention's appropriateness, intrinsic motivation is a critical element of play (Bundy, 2004). Findings suggest that when play is used as the means for facilitating an intervention, children are not only motivated to engage in the intervention (facilitating appropriateness); there are also functional benefits to the child's social participation in play.

Parents appreciated the expertise of the practitioner delivering the intervention and the care they displayed for their child. Occupational therapy practice frameworks include the therapeutic use of self within the therapeutic process, which involves empathy, and a client centred and collaborative approach to the delivery of services (e.g., American Occupational Therapy Association, 2020). Occupational therapy clients have also identified the therapeutic relationship as critical to the outcome of interventions (Cole & McLean, 2003), and finding therapy professionals that are the right ‘fit’ for their child is something that facilitates access of an intervention for parents with ADHD (Lu et al., 2022). The emotional connection that parents and children were able to make with the therapist facilitated open communication that led to bringing everyone onto the same page, and this trusting relationship between therapist, children, and parents likely facilitated the engagement in the interventions and was a critical precursor of the changes in understanding that placed everyone ‘on the same page’.

While parents did not explicitly state that development of their child's social play was an important outcome, their willingness to find ways to fit the intervention into everyday life and ongoing use of the intervention strategies point indirectly to the relevance of addressing play challenges of children with ADHD to the families involved. The outcomes of a treatment are a large factor in the treatment decision‐making process for parents of children with ADHD (Schatz et al., 2015), suggesting the motivation of parents within this study was also an indicator that parents perceived their child's functioning in social play an important outcome. Play has long been viewed as integral to children's cognitive, emotional, and social development (Parham, 2008); however, the value placed on unstructured play beyond preschool age appears to attenuate in favour of academic skills (Warash et al., 2017). Our findings suggest that this may not be the case for families of children who are challenged by social play. Parents of children with ADHD likely see ongoing support for social play as important during schooling years due to its importance for socialising with peers and developing friendships (Gifford‐Smith & Brownell, 2003), and the provision of appropriate and effective interventions for play for school aged children is critical for clinical practice.

The ecological validity of the intervention was partially supported by parents. While the experience of participating in the intervention had its challenges for some families, the enjoyment of the experience and perceived benefits counterbalanced the challenges for most families. Siblings participating as playmates overcame a barrier to participation for many families and increased the ecological validity of the intervention, as siblings are regular playmates of children with ADHD (Cordier et al., 2010). While the logistics of getting to the intervention sessions was challenging for some families, parents were able to integrate the strategies and language used within the intervention into their everyday lives during and after the intervention period. Ensuring psychosocial interventions fit to the context of the end‐users is critical to their appropriateness and eventual implementation (Lyon & Koerner, 2016), and the inclusion of siblings and implementation of strategies within the home support the notion that the intervention was compatible with participants' life circumstances.

Clear changes were noted by parents in their child's play skills, however the sustainability and continuation of those changes over time is unclear. Not all families implemented strategies when the social environment differed to those of the intervention (i.e., other playmates, or other settings) and parents noted that continued support beyond the intervention period would be needed to continue to see the changes they hope for in their child. Children with ADHD are at risk of long‐term adverse health and social outcomes (Bishop et al., 2019; Hechtman et al., 2016; Pelham et al., 2020), supporting the notion that continued support to develop protective factors to these adverse outcomes is needed to sustain change as children develop.

Ways to provide additional therapist support should be considered in in future development of the intervention. A co‐design approach to development with parents and children would maximise the likelihood of future adaptations being feasible, appropriate, and effective for families. The person–environment–occupation (PEO) occupational therapy model is well positioned to guide future development of the intervention (Law et al., 1996). This model emphasises that occupational performance is influenced by the interaction and fit between the person, their environment, and occupation, all of which are interconnected. Using the PEO model ensures the facilitators and barriers related to a child, their environment (including social aspects of adults and peers), and occupation of play with peers are identified. As the development of the intervention moves towards new settings (e.g., schools), considering the interactions between the child, their environment, and play is particularly important. From an occupational perspective, using this model also holds importance for occupational therapists seeking to support parents in their parenting role, which includes supporting their child's social skill development.

### 4.1 Limitations

Data from this study were gathered from a robust sample in terms of size; however, the purposive sampling frame potentially limited the generalisability of findings to the broader population of families of children with ADHD. The parents who participated in an interview were all able to participate in the intervention, which precluded insights into barriers to participation that potentially limited the intervention's appropriateness for some families. Interviews were conducted 1 month after the intervention to limit recall bias while still obtaining a sense of continuation of the intervention from families. A researcher independent of the intervention conducted the interviews to reduce the risk of social desirability bias within the interview process; however, the semi‐structured nature of the interviews may have limited a deep exploration of some parent's experiences.

### 4.2 Implications for practice and future research

Together, these findings suggest that the intervention is appropriate for children with ADHD and their families, and translation of the intervention into practice is indicated so that therapists can deliver their intervention in everyday practice. Findings suggest that therapist training in the delivery of the intervention will be critical to the implementation stage of this intervention approach. To ensure enjoyment of participation for parents and children alike, therapists must have strong expertise in the social play of school‐age children, the child‐friendly language used within the intervention, and strategies to engage with children in playful ways.

Further development of the intervention is also indicated to increase the appropriateness of the intervention for some children. Redesign of the home‐based components of the intervention (videos, manual) using a co‐design approach may increase the ecological validity of the intervention by ensuring children's engagement in the materials. More choice in ways to access the intervention would likely increase its ecological validity. The logistic challenges of attending a clinic and fitting the intervention into a busy schedule may be addressed through wider implementation of the intervention to broaden the availability of the approach to families across more locations, meaning families would have the option to access the intervention via a more manageable commute. Some parents may also prefer to mediate the delivery of the intervention at home, or access support by tele‐health, to overcome the logistic challenges of accessing a clinic site. Adaptation of the approach to the school setting may also increase access and benefits for some children by widening the circle of peers who are exposed to the intervention approach and strategies. Future research is indicated to adapt and support the effectiveness of alternative designs of the intervention (i.e., tele‐health and school based) and to ensure this clinic‐based intervention is effectively translated into occupational therapy practice through appropriate training.

### 4.3 Conclusion

Parents largely supported the appropriateness of the intervention, and it was viewed as beneficial to the children and made possible through the collaboration between the therapist, parents, and children. Parents indicated that their thinking was reframed by developing a new understanding of their child by observing their play. The therapeutic relationship between parents and the therapist was critical to the success of the intervention. Parents had to make sacrifices to find the time to participate in the intervention but viewed the interventions as worth the investment. Future adaptions of the intervention are needed to increase its ecological validity and to generalise the strategies to other social environments and playmates.

## AUTHOR CONTRIBUTIONS

*Study conception and design*: Sarah Wilkes‐Gillan, Reinie Cordier, Michelle Lincoln, and Anita Bundy. *Data collection*: Sarah Wilkes‐Gillan. *Analysis and interpretation of results*: Sarah Wilkes‐Gillan, Lauren Parsons, Dave Parsons, Natasha Mahoney, Nicola Hancock, and Yu‐Wei (Ryan) Chen. *Draft manuscript preparation*: Sarah Wilkes‐Gillan, Lauren Parsons, Dave Parsons, and Yu‐Wei (Ryan) Chen. All authors reviewed the results and approved the final version of the manuscript.

## CONFLICT OF INTEREST STATEMENT

The authors have no conflict of interest to declare.

## Data Availability

The data that support the findings of this study are available on request from the corresponding author. The data are not publicly available due to privacy or ethical restrictions.
